# Recognizing oneself in the encounter with others: Meaningful moments in systemic therapy for social anxiety disorder in the eyes of patients and their therapists after the end of therapy

**DOI:** 10.1371/journal.pone.0250094

**Published:** 2021-05-11

**Authors:** Rebecca Hilzinger, Javiera Duarte, Barbara Hench, Christina Hunger, Jochen Schweitzer, Mariane Krause, Martina Fischersworring

**Affiliations:** 1 Institute of Medical Psychology, Heidelberg University Hospital, Heidelberg, Germany; 2 Center of Studies in Clinical Psychology and Psychotherapy, Universidad Diego Portales, Santiago, Chile; 3 Millennium Institute for Research in Depression and Personality (MIDAP), Santiago, Chile; 4 Faculty of Social Science -School of Psychology, Pontifical Catholic University of Chile, Santiago, Chile; 5 Faculty of Medicine- Postgraduate School, University of Chile, Santiago, Chile; The Hong Kong Polytechnic University, HONG KONG

## Abstract

There is evidence that systemic therapy is effective, but there is little evidence about meaningful moments in systemic therapy in general, and none at all in systemic therapy for social anxiety disorders. Meaningful moments are one of the relevant research objects in change process research, as they contribute to a better understanding of therapeutic change.

**Objective:**

The objective of this study is to characterize and describe meaningful moments in the context of systemic psychotherapy, from the point of view of patients and their therapists, after the end of therapy. The therapy studied is a manualized, monitored systemic therapy for social anxiety disorder.

**Method:**

Semi-structured follow-up interviews were conducted separately with five patients and their therapists (N = 10). Methodological triangulation was used: Grounded theory was used to code the transcripts as described by Charmaz. Then the passages of the selected code “meaningful moment” were evaluated using thematic comparison, in line with Meuser & Nagel.

**Findings:**

Three categories involving meaningful moments were identified: (1) meeting other patients in group therapy session, (2) therapeutic resource orientation and (3) recognizing oneself in a diagnosis or pattern of behaviour. These categories emerged as contexts related to the occurrence of meaningful moments from a subjective perspective.

**Discussion:**

Meaningful moments seem to be consistently related to the therapist input and to specific interventions or settings, both from the perspective of the patients and the therapists. Two tandems each described a coincident moment. One central aspect of all 14 moments is that the patients and therapists described patients being able to acquire another outlook on themselves.

## Introduction

Over the last thirty years a great deal of research has been carried out in systemic therapy regarding its effectiveness and potential to produce change, with numerous publications, reviews and meta-analyses [1–3]. In particular, the efficacy of systemic therapy for treating anxiety disorders has been widely studied [4–9], coming to the conclusion that systemic therapy works.

However, until today, the leading question in psychotherapy research within systemic therapy has been whether a specific therapy is effective, focusing on verifying the effectiveness of treatment methods. This has been practically implemented in randomized controlled trials [1]. Validations of effectiveness are helpful for various reasons, but these findings leave therapy itself behind as an unopened black box, with less understanding of how and why therapy works [10,11].

Too little is known about the crucial mechanisms leading to successful outcomes, but if we want to know more about how therapy works, we have to examine processes rather than only comparing outcomes, in the tradition of change process research [10,12]. Bearing this in mind, many researchers have concluded that short extracts from therapies can give a better understanding of therapeutic processes. These extracts are known as “in-session events” [11,13].

## Research on meaningful moments in psychotherapy

Interest in the study of meaningful moments in therapy, also described in the scientific literature as “relevant events” or “significant episodes” in psychotherapy, can be seen as part of the development of the change process research (CPR) paradigm [10], which focuses on identifying, describing, explaining and predicting the processes that produce psychotherapeutic change [12]. This paradigm, which has contributed to a large body of research regarding change processes in psychotherapy has in large part been constructed based on the identification and exhaustive description of events which stand out from the therapeutic process in a significant way, in particular according to certain criteria or specific models. These events are considered relevant for change due to their potential to foster or hinder it, but do not follow certain principles or pre-established canons during the psychotherapy process, which makes them hard to measure. Even so, interest in studying them has grown and become more important over the years, giving rise to a large number of theoretical and empirical conceptualizations [14,15]. Because research into change processes in psychotherapy has developed from different perspectives, e.g. with and without reference to psychotherapy research, or in the context of specific theoretical and clinical models, various means of outlining, understanding and analyzing relevant episodes have been designed, using different methods to detect such episodes, such as the observation of videotaped sessions, transcriptions or in-depth interviews with the participants.

Duarte, Martínez & Tomicic [15] recently offered an overview of the main approaches used in research on change processes in psychotherapy. The authors distinguished and reviewed six lines of research into relevant events. The variety of the studies mentioned above becomes clear even within a single line of research. One aspect which these six approaches are described as having in common is that there are moments in the session that “stand out in clinical terms and do not occur all the time” (p. 261) [15]. The evidence also indicates that clients, therapists and external observers are all able to observe and identify in-session events, and that their assessments must be taken seriously as objective means of identifying them, as their experience of meaningful moments in therapy could offer a deeper understanding of these moments and how they are related to the therapeutic process and therapeutic change [13,15–17].

We assign our research to the avenue of “significant events in psychotherapy”, which started out with Elliott [16]. Elliott [16] described the patients as expert witnesses and central to understanding processes of change. This is in line with a first- and second-person perspective approach in the study of the psychotherapy process. He focused on the study of helpful and difficult events in therapy, as identified by clients. This type of research has spawned a wealth of studies prioritizing the client’s view of important moments during the psychotherapy process, e.g. [16,18]. Significant events have been studied mainly by reviewing the videotaped therapy with the patient and, on some occasions, with the therapist (separately), using the methodology of Interpersonal Process Recall (IPR). This procedure looks to provide the participants with memory recovery cues that can facilitate access to the session’s significant events. Another way in which significant events in psychotherapy have been studied is through retrospective narratives by patients or the therapist’s recollection of these events, e.g. [19,20]. Unlike the IPR method, with this approach the events are not studied immediately after the session, but when the whole psychotherapy process has been completed or at a particular point during the process, depending on the objective of each particular study [15]. Along these lines, Fornaro [11] introduced the concept of meaningful moments, emphasizing the subjective evaluation of an event for the entire narrative: what is important depends on who tells it and when it is told. This narrative can change over time.

As far as we know, there is very little research on meaningful moments in systemic therapies, and no studies have been carried out regarding therapeutic change processes or meaningful moments in systemic therapy for social anxiety disorder. Two projects on systemic couple therapy address meaningful moments. The focus of the first is on a theoretical conception of how research can be carried out on significant moments from a systemic perspective [21]. One key finding is that significant moments do not exist independently: the definition of “meaning” is always linked to the point of view of an observer, whether that is a therapist, client or scientist. Second, in the European research project “Relational Mind in Events of Change in Multiactor Therapeutic Dialogues,” biological, psychological and social system data are evaluated in relation to each other. Seikkula, Karvonen, Kykyri, Kaartinen and Penttonen [22] operationalized the relational mind in couple therapy and investigated the embodied attunement between two therapists and per couple. In addition, an individual Stimulated Recall Interview (SRI) took place within one day of the session. Participants were asked about their feelings, thoughts and bodily sensations while viewing video-recorded episodes from the session. As a result, the authors found that mutual attunement between the clients and therapists was observable in spoken dialogues as well as in bodily action [22].

Psychotherapy studies that address change processes often involve cognitive therapy treatment, e.g. [23–25]. This is not surprising, because cognitive therapy has been demonstrated to be an effective treatment for social anxiety disorder. In a case study, Penttinnen [26] focused on reflexivity as a component of change and its actualization as part of a cognitive-constructivist, short-term form of group psychotherapy for social phobia. Amongst other things, 13 conversational passages from 12 sessions with a client were analyzed by means of assimilation analysis and consensual qualitative analysis. Penttinnen reports “that progress in assimilation happened only when the client took a reflexive stance towards her inner experience or outer actions” (p. 1). The author concludes that the therapist’s responsiveness and sensitivity concerning the client’s reflexivity is crucial for the process.

In summary, it can be said that it is not yet known whether meaningful moments or even relevant events have been reported after systemic therapies for the treatment of social anxiety disorders or, if they have, what form they take. From a theoretical point of view, the main limitation of traditional ways of understanding relevant episodes is that they are recollected from a third-person perspective. In this perspective, an external observer identifies and analyzes relevant events by examining transcriptions or videos and focusing on the patient as the subject of change. Even though this is true, what seems to be missing is interaction and a first-person in-depth perspective. The majority of previous studies refer to a specific therapy session, whereas the focus of the research question rarely relates retrospectively to the entire psychotherapy process.

### Study objectives

The objective of this study is to characterize and describe meaningful moments in the context of systemic psychotherapy for social anxiety disorder, from the point of view of patients and their therapists, after the end of therapy. Our focus is on reconstructing the experience of in-session meaningful moments. That includes identifying and highlighting the characteristics, requirements and meaning of shared meaningful moments.

## Method

### Context of the study: Systemic psychotherapy for social anxiety disorder

The sample for data collection is based on a sub-sample of the German randomized controlled trial (RCT) “Comparing Cognitive Behavioral Therapy and Systemic Therapy for Social Anxiety Disorder” (SOPHO CBT/ST) [27]. As such, it is to some extent a qualitative follow-up study that relates to a subgroup of SOPHO CBT/ST. All the therapies that went into this study were monitored courses of systemic therapy for social anxiety disorder as defined by Schweitzer, Hunger, Hilzinger & Lieb [28]. This newly developed manual [28] originated in the context of an RCT [27] comparing cognitive behavioural therapy and systemic therapy for social anxiety disorder. All therapists underwent advanced training and received regular supervision, and extensive checks were carried out to ensure that they adhered to the manual [27].

#### Systemic therapy study manual

Systemic therapy follows Helm Stierlin and his colleagues’ work during the early 1980s [29]. The theoretical background was composed of post-Milan concepts (e.g. circular interviewing [30] and genogram interviewing), solution-focused language [31] and symbolic action methods [32], grounded in communication theory [33], social systems theory [34] and the concept of autopoiesis [35]. These resulted in interventions such as paradoxical interventions, externalizations, reframing or focusing on resources and strengths.

The manual is structured into four therapy phases [27]: “(a) generation of a joint understanding of the function of the presented symptoms and the communication patterns that stimulate the development and maintenance of these symptoms; (b) experimentation with changes: symptom prescription, paradoxical intention, exploration of social fear systems, externalization, deconstruction of shared belief systems, enactment of socially anxious interactions; (c) relapse prevention; and (d) refreshment and consolidation” (p. 4). The therapy mainly took place in individual sessions, some with significant others (e.g. family, partner, close friends) “aiming at (re-)establishing a solution- and resource-oriented understanding of the interactional processes within the affected social systems” and a special expanded group therapy session [36,37].

The manual was drafted for 26 therapy sessions. Therapy sessions were mainly 60 to 120 minutes long and the group therapy session lasted 180 minutes.

*Group therapy session*. In line with the therapy manual, the supervisor, six patients and their therapists met once in a group therapy session, which lasted three hours. The first part consisted in getting to know each other, and the fishbowl method, where patients and therapists discuss their experiences and wishes for further therapy. The second part was essentially the choral speaking method (pp. 292–294) [29], where patients’ belief systems are sung by the group until the patient begins to show altered reactions. Newly created sentences are converted into new choral parts. Participants who contributed to this category described experiences where they perceived similarities that they shared with others and differences that they became aware of. The patients perceived the shared similarities as providing relief.

A detailed description of the systemic therapy study manual can be found in Hunger, Schweitzer & Hilzinger [38].

### Inclusion/Exclusion criteria and recruitment

We used criterion sampling [39], selecting participants in the RCT [27]. Inclusion criteria required participants (1) to have already completed systemic therapy for social anxiety disorder (SOPHO CBT/ST) and (2) to agree to participate in the study. Exclusion criteria related to violations of the inclusion criteria.

Participation in this study was optional. In our outpatient clinic at Heidelberg University Hospital, we asked all RCT therapists (n = 7) and their patients a couple of months after the end of treatment whether they were both prepared to join. The therapists were individually invited to join the study by e-mail or telephone. When a therapist promised, we asked the corresponding patients in a second step by e-mail. We sent the patients and therapists a fact sheet with the contact details of the first and last author as well as information about the study objective, research questions and interview format. Though the first author was known to all participants in the study, the interviews were conducted by FONDECYT researchers. The interviews took place in the rooms that the therapists and patients knew well.

### Participants: Patients and their therapists

Five out of seven therapists showed interest in participating. This homogeneous sample consisted in five manualized systemic therapy cases: five patients and their therapists. All patients were diagnosed with SAD as the primary disorder (SCID). Four out of five patients were diagnosed with various secondary diagnoses (e.g. agoraphobia, depressive disorder). The patients were between the ages of 21 and 56 (mean = 40, SD = 15.07), including three women and two men. The duration of the therapy varied from 17 to 55 sessions (mean = 26, SD = 14.59). All patients successfully completed the therapy. The therapists were between the ages of 29 and 63 (mean = 45.8, SD = 13.88), including four women and one man. All therapists used the corresponding manual and attended the three-day manual training. Table 1 shows the sex and age of the patients and therapists arranged by couples, the number of sessions and the intervals between the end of therapy and the interviews. The therapists were systemic therapists (two with additional training in CBT), and all therapists were trained according to a study manual. They were supervised for one in every four therapy hours. The therapists reported having spent a range of years conducting psychotherapy from 3.5 to 38 years (mean = 11.8, SD = 13.14). The intervals between the end of the therapy and the interviews were between one month, three weeks (52 days) and one year, four months and over a week (499 days) (mean = 8.7 months or 264 days). The differences are because the therapies started at different times and the interviews took place over a similar period of time.

**Table 1:** Characteristics of the sample arranged by couple.

Therapy	Patient Gender/Age	Therapist Gender/Age	Number of sessions	Intervals between the end of therapy and the interviews (interview with the patient/interview with the therapist)
1	M (56)	F (57)	21	More than one year (380 days/393 days)
2	F (53)	F (40)	17	More than six months (211 days/ 273 days)
3	F (21)	F (63)	17	More than four months (135 days/ 140 days)
4	M (29)	F (40)	55	Almost two months (53 days/ 52 days)
5	F (41)	F (29)	20	More than one year and four months (499 days/ 499 days)

M, male; F, female.

### Interviews

The interviews lasted from 36 to 82 minutes (mean = 57.3 minutes, SD = 13.90 minutes). The interviews with the patients (mean = 63.8 minutes, SD = 15.93 minutes) took longer on average than the interviews with the therapists (mean = 50.8 minutes, SD = 8.7 minutes). The audio recordings were then transcribed and subjected to qualitative analysis.

### Analysis procedure

A qualitative approach was used to characterize meaningful moments in systemic psychotherapy, following the idea that researching into small units of a psychotherapy process sheds light on change processes [12]. The methodological procedure consists of two parts: data collection and data analysis. These are reported separately because they take place in different contexts, and different evaluation methods are used.

Data collection is part of the Chilean research project “Experiences of Success and Failure in Psychotherapy—Construction of a Comprehensive and Multidimensional Model of Psychotherapy” (FONDECYT project N° 1141179) [40]. The aim of this project was to generate a multidimensional conceptual model of successful and non-successful aspects of a psychotherapy process, based on the subjective experience of a variety of patients and their therapists. The data collection tool consisted in semi-structured interviews based on a semi-structured interview guide. We undertook several steps, described briefly as follows: (1) Translation of the interview guidelines (one set for patients and one for therapists) from Spanish to German, and of the FONDECYT code tree. A bilingual researcher translated the interview guidelines into German and two German researchers carefully checked their cultural adaptation. The interview guidelines focus on the therapists’ and patients’ experiences of the therapy process. The opening instructions for patients are “Tell me about your experience during your psychotherapy treatment. I would like to pick up your general impressions, whatever comes to mind.” and the question for therapists is “I would like to ask you about the therapy process with your patient. How was that experience for you?” Both sets of interview guidelines [41] focused on the six topics (a) Diagnosis and notions of illness (e.g. “What moved you to seek help?”); (b) Therapy expectations (e.g. “In what way did you think therapy could help you?”); (c) Therapeutic relation (e.g. “How did you feel with him/her?”); (d) Significant moments and interventions (e.g. “Was there any moment you saw as significant?”); (e) Outcomes (e.g. “How do you evaluate the therapy process?”); (f) Termination process (e.g. “How did therapy come to an end?”). Throughout the interview, both the patient and the therapist were encouraged to share anything they felt had been relevant for them or for the change process and describe specific events that had occurred during the therapy as examples of their reflections. (2) Selection of the sample based on the participants in the RCT [27], following criterion sampling [39], and meeting the criteria of homogeneity. (3) Conduction of the interviews by two of the authors. These were conducted separately with the patient and therapist. (4) Checking credibility and epistemology: the patients and therapists were asked at the end of the interview whether they wanted to add any information (“Would you also like to add something that we have not talked about yet?”). (5) Coding the units of meaning in the interview transcripts following the principles of Grounded Theory [42], building up on the code tree (combination of deductive and inductive coding). The data from this study enrich FONDECYT and contribute to theoretical saturation. The 12 main categories of the code tree are (a) global evaluation of psychotherapy, (b) characterization of the therapeutic encounter, (c) context of the therapy, (d) descriptors for the therapy progression, (e) forms of therapeutic work in past/current psychotherapy, (f) start of psychotherapy, (g) motivation/expectations regarding psychotherapy/description of the problem, (h) perception of other treatments, (i) impressions of psychotherapy, (j) results of psychotherapy, (k) events leading up to current psychotherapy and (l) therapist’s experiences related to their therapeutic actions. (6) Ensuring interrater reliability: the data analysis took place in alternating two-person teams under the detailed advice of a FONDECYT researcher. After each step of the data analysis, we discussed the findings in the group.

The data analysis relates exclusively to the passages in the interviews that were coded with the code “meaningful moment” in the first part. This sub-code is part of the main category (d) descriptors for the therapy progression. The aim of the data analysis is to characterize and describe meaningful moments. We undertook several steps, described briefly as follows: (1) Removing all passages from the transcripts that we had coded with “meaningful moment”. (2) Structuring similarities and differences between the extracted codes following a special type of content analysis: Meuser and Nagel´s [43,44] thematic comparison method, which follows the principles of inductive coding. The intention is to identify similarities and differences (“inter-individual similarities”). The resulting category system answers our research question. (3) Ensuring interrater reliability: Two researchers implemented each step of the evaluation independently of one another and then contrasted them together. The two went on to critically discuss the findings with two other researchers.

ATLAS.ti (https://atlasti.com) was used to manage the qualitative analysis. With regard to the reporting guide, we followed the standards for reporting qualitative research proposed by O’Brien et al. [45].

### Approval by the ethics committee

SOPHO CBT/ST was approved by the Ethics Committee of the Heidelberg Medical Faculty (S-190/2014) and registered with the U.S. National Library of Medicine (ClinicalTrials.gov): #NCT02360033. The study follows the principles of the Declaration of Helsinki. Before the interview started, we discussed a consent form with each participant. Every participant gave written consent to the inclusion of material pertaining to themselves, acknowledging that they cannot be identified via the paper and that they were fully anonymized. Essential contents concerned the confidentiality of the interview, recording with an audio recording device, the use and storage of data, the possibility to withdraw from participation at any time without indication of reasons and contact details for inquiries.

### Researchers’ reflexivity

The study involved cooperation by Chilean and German researchers. The researchers shared an interest in process research, and especially in the exploration of significant moments. The Chilean researchers had expertise in the tradition of process research and qualitative methods, in particular from many years of research by the study group on Psychotherapy and Change led by Mariane Krause since 2002, constituting a research line in MIDAP (midap.org) since 2015. The German researchers were partly involved in the RCT and interested in a qualitative follow-up study. The data collection took place in Germany. There were six participants in the data production and analysis: MF from Chile, a researcher in psychotherapy and experienced clinician with a constructivist psychotherapeutic orientation and RH, a doctoral student and clinician from Germany with a systemic orientation, along with EH and BH, Bachelors students in psychology, and AB and MZ, Masters students in clinical psychology, all four of whom were interested in qualitative methodology and involved in this study as trainees. JD had therapeutic training in cognitive constructivist orientation and previous experience researching with qualitative methods in the psychotherapy process and significant moments. With her expertise on significant moments, JD contributed to the current state of research and to the discussion of the results.

## Results

The data were derived from the interview transcripts (N = 10). After the qualitative evaluation, we identified 14 meaningful moments (codes) assigned to three categories (Table 2). These codes were organized according to their similarities, which were then grouped into categories. The description of the results includes a specification of each category and meaningful moment. The numbers in brackets indicate the pair from whom the statement came.

**Table 2:** Categories and meaningful moments.

Categories	Meaningful moments	Consequences	Therapy
**1: Meeting other patients in group therapy**	(1) Seeing yourself reflected in another patient [P1] (2) “we are all in the same boat”[P2] (3) Insight that others do not perceive one’s inner processes [T5] (4) Others’ success arouses hope for their own progress [P3] (5) Realizing that you are less affected than the others [P5] (6) Interventions in group settings can activate different things in different patients [T4/P4]	• Changes your self-image • Changes motivation for the therapy	1, 2, 3, 4, 5
**2: Resource orientation**	(7) Unfamiliar praise leads to emotional release [P1] (8) Exercising willpower during an exercise [P1/T1] (9) Feeling the therapist’s trust and bringing yourself to do something [P1] (10) Perceiving the therapist’s joy and then also being happy about something [P2] (11) Seeing your own resources in a sketch [P1] (12) Recognizing the positive change in the patient’s self-image [T1]	• Strengthens the therapeutic relationship • Helps to overcome fear • Changes your self-image and self-esteem • Leads to display of feeling	1, 2
**3: Recognizing oneself in a diagnosis or pattern of behavior**	(13) Recognizing yourself in a diagnosis [P5] (14) Recognizing your own patterns [T5]	• Rewriting one’s own life story changes one’s self-image • Self-diagnosis generates motivation • Becoming more able to act	5

P, patient; T, therapist.

### Category 1: Meeting other patients in group therapy

Statements from patients referring to meetings, observations or perceptions in group therapy are summarized in the category “Meeting other patients in group therapy.” Participants who contributed to this category described experiences where they perceived similarities that they shared with others and differences that they were aware of. In meaningful moments (1) to (3), participants perceived shared similarities as providing relief. Meaningful moments (4) to (6) referred to differences that were perceived in the group therapy session.

#### (1) Seeing yourself reflected in another patient

One patient reported on an encounter in group therapy with a patient of a similar age, who resembled him. After a few weeks, they met again, identified other similarities, and shared their achievements and progress so far:

*[…] then everyone introduced himself and [a man] sat next to me who said that I could be his brother, because the same applied to him. After a few weeks, we met again privately, talked and found many parallels, including the same causes of these phobias. […] Yes, exactly, and there we talked about the successes that the whole thing had already brought, what we’d already dared, what had become better and yes, that was quite interesting then. (P1)*.

#### (2) “We are all in the same boat”

One situation as part of a group therapy session was meaningful to another patient. The patient described a moment when the others sang her sentence.

*The group therapy session as a whole left an impression on me. It helps a lot. […] Then one person from the group came to me. She copied me and modeled my reaction. And then another came and one more. Then we were almost stronger than the other side. And then relief came to me. I can’t describe what happened right now, but that was a significant moment. (P2)*.

#### (3) Insight that others do not perceive one’s inner processes

A therapist described how her patient received feedback from other patients that she appeared self-confident. The therapist shared that perception. In her opinion, the advantage of the group therapy session was that those involved knew that all of the patients were affected by social anxiety and could therefore say “hey, we do not even notice that you are anxious.” Otherwise, the patient hid her social anxiety and could not get any feedback. The therapist first understood how anxious the patient was in many situations:

*Yes, it was only then that she really realized it […] I don’t know if she felt self-confident, but at first it was a “wow, nobody can see it.” Above all, it was a relief. (T5)*.

#### (4) Others’ success arouses hope for their own progress

One patient reported that she was more positive after the group therapy session and had hopes that she would feel better too. She had the impression that other patients who had already completed more hours of therapy could cope with their social anxiety better:

*It was definitely like that, it clicked with me a little because I realized that it definitely happens more often than you imagine […], you’re not just alone with it, other people are like that too, even if it’s different, but somehow they all have the same irrational thoughts and that it does not matter what social milieu you come from or what age you are, but that in principle it can influence everyone, yes. […] But it was definitely something that I thought, “so they can’t all be wrong now, it seems like [the therapy] helps a bit.” (P3)*.

#### (5) Realizing that you are less affected than the others

Meeting other patients left an impression on one patient, as she found that she was much less burdened than the other patients. She described being very excited about her insight that she had quite a harmless social phobia compared with others. Although she was not keen on such group settings, she found it extremely helpful:

*And I do not remember exactly what it was about, but I felt like “Oh God, I’m going red,” and the others said “no, not at all.” And I thought “Okay, weird. I can’t believe it.” And it was the same with me, the other way round. People who said, “Oh God, I thought I’d die” and I hadn’t noticed. And that’s the kind of feedback that’s really important to me. Nobody realizes that you are agitated. I thought that was very good. (P5)*.

#### (6) Insight that interventions in group settings can activate different things in different patients

One therapist characterized the result of a group therapy session as a meaningful moment. She had offered the same intervention to three patients with the same diagnosis. One participant subsequently experienced a drastic change. For him, the intervention left such an impression that he decided the night after the therapy session to break off his studies, which he had started for the sake of his father:

*[…] I found it impressive: It is the same intervention for three people with the same diagnosis. Two patients do everything as before. […] He really made a break. (T4)*.

Her patient describes a meaningful moment in the same group therapy session. From his narrative it becomes clear how looking at a picture card revealed extensive changes.

*That was a year ago, just before Christmas. […] The negative picture especially triggered so much, and then there was quite a turnaround. […] It became clear to me that I am hiding and that I am not showing myself. Not even to myself. For me it was the idea in group therapy of taking a step out from behind the tree. Then our agreement by the next therapy session was to take a step in front of the tree. […] Then I first showed the other side of my face. To me. To my parents. And then decided what was overdue. […] At night I was upset, cried a lot, like almost never before. […] I looked in the mirror and started to cry. Because I acknowledged how bad I am. (P4)*.

### Category 2: Therapist’s resource orientation

This category summarizes statements from patients and therapists referring to a therapist´s attitude of resource orientation. Meaningful moments (7) to (10) are statements the therapists made when talking to her patient. Meaningful moments (11) and (12) are experienced during a resource-oriented exercise.

#### (7) Unfamiliar praise leads to emotional release

One patient told of the therapist’s praise in the first therapy session, which he considered to be significant for the further course of the therapy. The therapist wanted to know a lot about his family and children and then told him that she thought he was a good dad. That increased his self-confidence and pleased him. At the same time, he was ashamed and it was initially unpleasant for him. Praise of that kind was unusual for him. From then on, it was no longer unpleasant. He emphasized that he found it very important that she had managed to cast aside his shame.

*I burst into tears when she praised me so much at the end of this session because it was so unusual for me. It was soothing but very emotional. […] On the one hand, it was nice […] the feeling of pride […] and on the other hand it was an uncomfortable feeling. (P1)*.

#### (8) Exercising willpower during an exercise

One patient experienced a significant moment in the therapy session as he practiced a speech for his job in front of the therapist and two interns. The patient, a senior leader with a lot of customer contact, had to exercise willpower during the exercise:

*There was another moment like that […] and that took me a lot of willpower though it was only a speech in front of my therapist and two interns […] put me on a chair and made me do that. (P1)*.

His therapist remembered the same moment. She also thought it was significant that the patient overcame his anxiety and received positive feedback.

*I had the impression he could do it, it was OK what he did, he didn’t stutter, could express himself and hold the tension. That feedback was something extremely valuable for him. […] He was prepared, he was mentally prepared for this […] and he had the courage to stand on a podium at the end of the exercise. (T1)*.

#### (9) Feeling the therapist’s trust and bringing yourself to do something

The patient and his therapist spoke about the route the patient wanted to drive to go on vacation. The question was whether the patient was going to go over a pass or drive through a tunnel. Feeling the therapist’s trust was so important to the patient that he brought himself to drive through a tunnel, a very frightening act:

*And then she said, “You drive through the tunnel. You can do that. You don’t have to worry about anything” and then I drove through the tunnel. On the way there with a slightly queasy feeling and on the way back it wasn’t that far anymore. Nobody wants to get stuck or have an accident in a tunnel. The statement by my therapist was very important. Simply the confidence […] simply with the full conviction and certainty that I can do it. That strengthened my self-confidence, challenged me, like “Now I’ll show her that I can do it.” And then the first question was, “And did we drive through the tunnel?” And I said, “Yes, sure, don’t you trust me?” (P1)*.

#### (10) Perceiving the therapist’s joy and then also being happy about something

A patient explained that it is difficult to define one meaningful moment. She often became aware of the meaning of some therapy sessions with hindsight. She described the therapist’s shared enjoyment and confidence as important.

*Sometimes I thought the whole session was significant, not just a single moment. […] Sometimes later, the week after, a sentence or topic returned to my head. The aftereffect or the process. Immediately after the therapy session, I couldn’t say what exactly the moment was. […] What was significant was her joy, that I hadn’t experienced before from other people. And the attention. That already did a lot. I was taken seriously. […] Once she encouraged me to take a course. I did the course. She was so happy about it and then I could internalize the joy. I can look forward to my self-created successes. Yes, exactly, I think that was meaningful. Previously, I still had voices in my head that said: you are not allowed to do that or you are not worth it. With her joy, I was able to switch off these thoughts. (P2)*.

#### (11) Seeing your own resources in a sketch

Seeing his own resources at a glance in a sketch was meaningful for another patient. His therapist and the patient made a drawing of a male and wrote all of his resources by it:

*Yes, we once made a kind of little man where all this was written down, what I achieved in my life. […] that I always have to keep this in mind when I doubt myself again. This man just reminds me. She gave it to me at the end and I put it on my desk so it keeps reminding me. (P1)*.

#### (12) Therapist: Recognizing the positive change in the patient’s self-image

One therapist considered a patient’s change of perspective while visualizing his own resources as a significant moment. During the intervention he could see what he had already achieved in life. That allowed him to look at himself in a benevolent way:

*I really felt that something was happening there. That was with this male. Even if it was more like a cognitive process. I had the impression that he was able to realize what resources he had […]. A radical change happened. I can’t say why. It was a movement. […] A change of perspective took place. Maybe it’s something he developed a bit of pride in. […] That was such a moment when he was able to say “Actually, I am doing quite well.” (T1)*.

### Category 3: Recognizing oneself in a diagnosis or pattern of behavior

This category combines two descriptions in which the patient recognized herself in the diagnosis of social anxiety and in patterns of behaviour described by the therapist.

#### (13) Recognizing yourself in a diagnosis

Recognizing herself in a diagnosis was meaningful for one patient. At the beginning of her therapy process, the patient went through some “aha moments” during the diagnostic interviews. Later, these were repeated. By seeing correlations for herself, she was able to act differently.

*Just at the beginning […] the aha moments […] left such an impression on me, when I realized: I’ll fit in there! That’s exactly my problem! And it worked when I tried things and those were the aha moments. And my therapist offered just the right ideas for a solution. That was great. […] So first of all in these interviews […]. There were a lot of times when I found myself again. And in structuring, dividing things into areas and spotting: What are my symptoms? And what are the reasons? Those were real aha moments, because I thought: Boy, I never thought it was connected with a fear like that. And that already began in my childhood. […] The moment I understand the context, I’ve already changed something. (P5)*.

#### (14) Recognizing your own patterns

One therapist believed that it was significant to identify patterns together. She described a systemic intervention called Problem-Solution Balance, see [28,46]. In this four-field scheme, the patient’s possible inner feelings of ambivalence between changing and staying the same can be considered. She used the intervention to develop an understanding of how the patients’ behavior at work and in her partnership could be related to social anxiety.

*I guess that the true turning point in the therapy was when I had one intervention, because […] one thing did not fit. The theory was that she accumulated anger at work and “unloaded” it at home. But then it escalated on vacation and that didn’t fit. I came up with the idea that the trouble might be whether she decides something for herself or she has to do what others ask for. […] After doing some experiments while on vacation, she realized she had an influence on herself. (P5)*.

## Discussion

The objective of this study was to characterize and describe meaningful moments in the context of systemic psychotherapy, from the point of view of patients and their therapists, after the end of therapy. A different number of themes were coded in the interview transcripts. While six themes were coded in one therapy process (Therapy 1), in two other therapy processes (Therapies 3, 4) a single moment was coded as meaningful. In Therapy 3, only the patient formulated a meaningful moment (4) “Others’ success arouses hope for their own progress” (P3). And in Therapy 4, P4 and T4 exclusively formulated a matching moment (6) “Interventions in group settings can activate different things in different patients” [T4/P4]. This finding that significant therapy moments are not inevitably captured is also reported in the research literature [15].

One conclusion of this work is that meaningful moments have been reported consistently related to therapists’ input and to specific attitudes, techniques or settings from the perspective of both the patients and the therapists. It can be stated that it was the offer of a group therapy session and the resource-oriented attitude of the therapists which clients and therapists saw as a particularly significant experience afterwards. The findings reveal consistent links with existing studies—for example the report on a patient who found the verbalized feedback from the group on her blushing important [47]. Acceptance of one’s own experience, whose relevance becomes clear [47,48], was not explicitly addressed, but we hypothesize that the experience of having feedback from the group resonates with our results.

Interestingly, in the case of Category 1 “meeting other patients in group therapy”, it is–with one exception–not the deconstructed beliefs that the leading therapist has worked out with each client, but the setting itself which gives rise to meaningful moments. In the statements by the clients and therapists it becomes clear that the clients’ group sessions made special experiences possible. Studies on Cognitive Behavioral Therapy have shown that exposure to stressors is effective, and we hypothesize that the group setting may have been some form of exposure–especially for patients suffering from social anxiety. The group context itself is very challenging for people who suffer from social anxiety disorder, because the anxiety manifests in the presence of others. It is thus not surprising that the group context plays a role here, as social anxiety is experienced with others or in groups. Meaningful moments seem to be related to changes in the patients’ self-image and self-esteem which enable them to overcome the paralysis that is often experienced in social anxiety. One of our hypotheses is that the altered perspective on themselves that was made possible in the group therapy session could also have been made possible through the preparations for the group therapy sessions. The therapists encouraged their clients to attend the group therapy session and were present themselves. This could have given clients security and trust. These changes seem to be related not only to how the group sees them but also to how the therapists see them.

The descriptions of the encounters are reminiscent of Mead’s conception of the development of the self [49]. According to this theory of identity development, individuals continually compare others’ ideas about them with their own ideas about themselves and thus develop their identity. One interesting aspect here is that this is not a comparison with any random other, but specifically with others who suffer from similar burdens. A common feature of the statements in this category is that all the clients and the two therapists positioned the patients’ burden in relation to that of others and gave the relationship a positive connotation.

In the case of Category 2, “resource orientation”, the corresponding therapeutic attitude can be seen as a common aspect of the statements. Most of the statements are spontaneous, benevolent and optimistic feedback from the therapist, which was not planned but can be linked to the basic attitude of a systemic therapist. In this category, (11) “seeing your own resources in a sketch” and (12) “recognizing the positive change in the patient’s self-image” are also two meaningful moments that were explicitly named in connection with a resource-oriented technique. The current state of research is that resource orientation and activation are considered to be one of the most important effects of psychotherapy across various disorders and therapy schools [50]. Accordingly, studying meaningful moments in the context of resource orientation improves our understanding of the mechanism of change underlying them.

At first glance, Category 3 differs from the other two categories in terms of the number and content of the moments. However, in the following it is shown that both of the moments assigned to this category can be viewed as closely related to other meaningful moments. “Recognizing oneself in a diagnosis” (P5) is used to code a statement in which the client describes feeling relieved that her burden has a name and a diagnosis, and that there are many other people who suffer from social anxiety disorders. This is similar to the meaningful moments [1] “Seeing yourself reflected in another patient” (P1) and [2] “We are all in the same boat” (P2). In the group therapy session, P1 and P2 relate to fellow patients and thus perceiving oneself as part of a group, whereas P5, in the individual therapy setting, relates indirectly and theoretically to all social anxiety sufferers. The client feels part of a group when she learns that there is a diagnosis for her condition, and various types of therapy. Following a psychodynamic explanatory model, this shared aspect could be interpreted as showing that a feeling of social belonging fulfilled a genuine human need for social belonging. For people suffering from social anxiety, this could in turn be particularly significant, as their suffering is particularly evident in interaction with other people and makes it hard for them to feel social belonging. The remembered meaningful moment “Recognizing oneself in a diagnosis” (P5) is also notable in that it was experienced in the context of systemic therapy, which is not traditionally carried out based on any specific disorder and does not make any diagnoses. Within the German systemic therapy community, there has been intense debate for many years on whether systemic therapy should target specific disorders, as this would affect the method’s recognition under social security law [51]. This is a special case, as the systemic therapy sessions were preceded by a complex diagnostic process based on the Structured Clinical Interview for DSM [27,52].

The moment “Recognizing your own patterns” (T5) described by one therapist goes back to an intervention including the patient’s patterns and at the same time a positive reframing of the “problem”. Reframing is a typical systemic intervention that looks for the good in a bad situation and is resource-oriented. The story told about T5 differs from the moments reported in Category 2 in that its focus in the interview is more on the description of the “problem” than on the “solution”. The moments in Category 2 necessarily theoretically imply that the resource-oriented interventions took place due to a “problem”, but with the difference that this “problem” was not in the foreground in the interviews, or possibly also the therapy sessions.

If we look at the meaningful moments identified by the patient and the associated therapist, we are given the impression that two tandems were describing the same situation. Two months after the end of therapy, P4 and T4 describe the result of a group therapy session in which a patient “steps out from behind the tree” in accordance with the chosen image card and makes drastic decisions about his life. P1 and T1 even reported the same situation a whole year after the end of therapy. In the role play, the patient gave a speech that he was to deliver to a large group of people in the near future. The therapist’s encouraging feedback even got him on a podium during the third run. Significantly, they both mention the therapist’s supportive words. This suggests that meaningful moments can be captured well with the interview method used, because, as this example shows, the patients and therapists do not just describe any random moments, but rather carefully selected and sometimes identical aspects. With reference to corresponding studies, Altimir et al. [53] summarize that “coincidence between participants on exact in-session events is low”. We have the hypothesis that P1 and P4 could have remembered the same moments because both moments were tried out in the therapy situation in their imagination and later actually implemented by both patients outside of the therapy situation: P1 actually delivered the rehearsed speech in front of an audience and P4 decided to stand up for himself and the fact that he would drop out of his studies. This distinguishes these two moments from the rest of the moments that do not relate to concrete actions, with the exception of another moment expressed by P1. After P1 [9] felt the trust of his therapist, he actually drove his car through a tunnel. The remaining moments seem to be less concrete and more abstract. More research is needed to make further statements about these hypotheses. Interesting questions could include whether the matching moments were already addressed in the therapy, or whether the answer would have matched if the interview had been held at a different time.

One final result is an essential commonality shared by the 14 meaningful moments: the fact that the patients and therapists described patients being able to acquire another outlook on themselves. “How I look at myself changes” is more or less a central aspect and is almost a reframing as understood in systemic theory. This is reminiscent of Heatherington et al. [1], who commented “For example, in family therapy, one principle of change might be helping clients achieve a new, more constructive perspective on their interpersonal conflicts, with different approaches using various strategies […].” (p. 352). From this study it is possible to state that one important principle might also be helping patients achieve a new, more constructive perspective on themselves.

Numerous systemic methods are described in the manual that relate to changes in perspective–such as genogram interviews, circular questions or working with internal systems [28]. Based on the adherence ratings in the RCT study, indicating that the systemic therapists frequently demonstrated adherence to the manual [27], it can be assumed that changes of perspective were repeatedly offered by the therapists and thus practised. In this respect, the hypothesis can be formulated that adopting a meta-perspective is of particular importance in systemic therapeutic approaches. This must first be checked in comparison with other theoretical approaches. To the best of our knowledge, since this study is one of the first studies on significant moments in systemic therapy processes, no links to comparable research results can yet be established.

### Implications for clinical practice

Heatherington et al. [1] assert that “Change process research has direct relevance to clinical practice.” Our results give ideas and orientations for clinical practice, specifically for systemic therapy settings, but the point is not to give instructions on how significant moments can be brought about. Meaningful moments can be potential levers for therapeutic change and therefore relevant to practitioners (p. 157) [21]. For clinical practice, it might be important to regard the group therapy setting not only in an inpatient context, but also in an outpatient setting.

### Implications for further research

Studies on significant events using different therapeutic orientations have repeatedly shown that the presence of these events are related to symptomatic outcome. These studies have also shown common themes among them, such as awareness of feelings and thoughts in the context of the therapeutic relationship, problem solving, moments of insight, and behavioral change [54–59]. Our study helps understand significant events in the context of systemic therapy and shows that these aspects can also be found in this form of therapy. As shown in the findings, moments of insight and behavioral change seem to be very important for this group of patients and whether they are able to see themselves differently because of how their therapists and the group sees them, and act despite their paralysis. Another interesting finding is that these moments are also experienced in a group context, which opens up a new possibility of understanding significant events not only in a dyad but also within a larger group—and seeing how these events take place.

It is also noteworthy that the meaningful moments recognized by the patients and therapists in this study seem to take place in the first third of the psychotherapy process. In one case, the described meaningful moment even occurred during the first session. This finding is consistent with other works such as that by Comninos & Grenyer [60], suggesting that successful therapy can already be recognized from early sessions.

It could be revealing to explore the setting of the group therapy session (meeting other patients and their therapists) as part of (systemic) therapy, in terms of research on impact factors: What impact factors arise from this exceptional setting? Our hypothesis is that the group therapy session addresses all of the four formulated factors of “resource activation,” “motivational clarification,” “problem activation” and “mastery” as described by Grawe, Donati & Bernauer [61]. Jensen et al. (p. 306) [47] posit that acceptance by the group is “considered as an important non-specific therapeutic factor in group therapy (Yalom, 1975/1995).” More research on mechanisms of change needs to be conducted.

### Limitations and strengths

While traditional process studies focus on the patient’s perspective, we consider it a strength also to have recorded the therapist’s perspective. As shown by the present study, we found consistent information on meaningful moments in the statements by the patients and therapists. A rare element of this study is the large gap between the therapies and the interviews. The fact that we did the follow-up interviews about eight months after the therapy ended means that the patient may have a broader perspective on possible personal changes triggered by these specific attitudes and techniques at that specific moment. Finally, one finding that we think deserves particular comment is the researchers’ reflexivity. The combination of expertise in the tradition of process research on the one hand and expertise in systemic therapy and the conducting of a study on the other hand enabled the present study to take place.

A methodological limitation in relation to the current state of research is the investigation of meaningful moments “on one channel” using the interview transcripts. Research shows that emotions can be made visible or represented in writing, for example through the use of conversation analysis, e.g. [62]. However, the importance of the difference between a transcription and the spoken word is often overlooked in interview-based research. Nevertheless, it was not only the coded transcripts that formed the basis of our data analysis: we constantly listened to the audio recordings of the interviews during the data analysis. When coding the transcripts, we found that it was essential to know and listen to the audio recordings of the interviews. Those who were not involved in the transcription and evaluation repeatedly questioned individual moments as to whether they were actually significant. In retrospect, we come to the conclusion that it is essential to hear the audio recordings when evaluating the data. Some moments may not appear significant when a description is read. When researchers listen to the audio recordings, the pauses, stresses, changes in speed when speaking and changes in volume make it clear that an unusual moment has been described, see [62]. Even if we did not use a complex transcription system, we noticed that we coded meaningful moments as such when they sounded like a spontaneous idea and differed from the other passages in terms of their louder tone, more rapid speech and fewer pauses.

This study has limitations and the findings should be interpreted with caution. They are based on a small number of clients and therapists; even so, these were able to provide rich qualitative data which are consistent with previous research findings in this field [15,47,50,53]. According to Stiles [63], the question about the generality of the findings for this type of study is about the question of transferability of the findings, which is stated as an alternative understanding of generality. Future studies may transfer the finding that meaningful moments might also be related to meetings with other participants in group psychotherapy for social anxiety disorders. It might be helpful for research into meaningful moments to focus not only on the level of individual participants but also on meetings by participants and to enable these meetings in further study designs. Furthermore, the analysis procedure used proved to be favorable and could be transferred to future studies.

The present study may be complemented with observational approaches. Generic Change Indicators (GCI) [64], for example, involves moments of change being captured by independent observers and assigned to one of 19 indicators based on videotaped therapy sessions. Another example is Brief Structured Recall (BSR) [65], which first uses the Helpful Aspects of Therapy (HAT) questionnaire [66,67] generally by the patient to identify significant events. While these sequences are watched together with the patient or therapist on video recordings, further procedures such as free-answer descriptions or rating scales can be used.

A different view of the data would be provided by studies of how meaningful moments appear over time from one therapy session to another. In a case study following on from this study, the evolution of meaningful moments during the course of therapy is examined.

It would be very interesting to replicate the study after the end of systemic therapy with families or couples and more closely examine the perspectives of the therapist and several family members or a couple. A deeper understanding of what patients and therapists experience as significant could make therapy offers even more helpful in the future and ultimately enrich the training of psychotherapists.
